# The Role of Motivation in Cognitive Reappraisal for Depressed Patients

**DOI:** 10.3389/fnhum.2017.00516

**Published:** 2017-10-31

**Authors:** Xiaoxia Wang, Xiaoyan Zhou, Qin Dai, Bing Ji, Zhengzhi Feng

**Affiliations:** ^1^Department of Basic Psychology, School of Psychology, Third Military Medical University, Chongqing, China; ^2^Department of Clinical Psychology, Chongqing City Mental Health Center, Chongqing, China; ^3^Department of Psychological Nursing, School of Nursing, Third Military Medical University, Chongqing, China; ^4^Department of Radiology, Southwest Hospital, Third Military Medical University, Chongqing, China; ^5^Department of Behavioral Medicine, School of Psychology, Third Military Medical University, Chongqing, China

**Keywords:** cognitive reappraisal, major depressive disorder, model of the cognitive control of emotion (MCCE), behavioral inhibition system (BIS), behavioral activation system (BAS)

## Abstract

**Background:** People engage in emotion regulation in service of motive goals (typically, to approach a desired emotional goal or avoid an undesired emotional goal). However, how motives (goals) in emotion regulation operate to shape the regulation of emotion is rarely known. Furthermore, the modulatory role of motivation in the impaired reappraisal capacity and neural abnormalities typical of depressed patients is not clear. Our hypothesis was that (1) approach and avoidance motivation may modulate emotion regulation and the underlying neural substrates; (2) approach/avoidance motivation may modulate emotion regulation neural abnormalities in depressed patients.

**Methods:** Twelve drug-free depressed patients and fifteen matched healthy controls reappraised emotional pictures with approach/avoidant strategies and self-rated their emotional intensities during fMRI scans. Approach/avoidance motivation was measured using Behavioral Inhibition System and Behavioral Activation System (BIS/BAS) Scale. We conducted whole-brain analyses and correlation analyses of regions of interest to identify alterations in regulatory prefrontal-amygdala circuits which were modulated by motivation.

**Results:** Depressed patients had a higher level of BIS and lower levels of BAS-reward responsiveness and BAS-drive. BIS scores were positively correlated with depressive severity. We found the main effect of motivation as well as the interactive effect of motivation and group on the neural correlates of emotion regulation. Specifically, hypoactivation of IFG underlying the group differences in the motivation-related neural correlates during reappraisal may be partially explained by the interaction between group and reappraisal. Consistent with our prediction, dlPFC and vmPFC was differentially between groups which were modulated by motivation. Specifically, the avoidance motivation of depressed patients could predict the right dlPFC activation during decreasing positive emotion, while the approach motivation of normal individuals could predict the right vmPFC activation during decreasing negative emotion. Notably, striatal regions were observed when examining the neural substrates underlying the main effect of motivation (lentiform nucleus) and the interactive effect between motivation and group (midbrain).

**Conclusions:** Our findings highlight the modulatory role of approach and avoidance motivation in cognitive reappraisal, which is dysfunctional in depressed patients. The results could enlighten the CBT directed at modifying the motivation deficits in cognitive regulation of emotion.

## Introduction

Emotional disturbance figures prominently in major depressive disorder (MDD), with anhedonia and negative affect as key psycho-pathological dimensions. Emotional dysfunction predicts the severity of symptoms, non-response to antidepressant treatment and non-remission in depression (Vrieze et al., 2013). Theoretically, it is posited that compromises in cognitive control of emotion may be central to the psychopathology of major depression (Ressler and Mayberg, 2007; Disner et al., 2011). According to the integrated model of cognitive control of emotion (MCCE) (Ochsner et al., 2012), the most commonly studied exemplar of cognitive control of emotion is reappraisal, which is typically steered toward weakening or changing the emotional response to a stimulus by reinterpreting its semantic meaning. Recent functional neuroimaging studies have mapped the brain systems that support reappraisal of emotional stimuli, which increases activation in executive control regions and decreases activation in subcortical regions such as the amygdala (Kanske et al., 2012; Perlman et al., 2012; Dillon and Pizzagalli, 2013; Smoski et al., 2013). In MDD, instructed reappraisal strategies instantiate hyper-/hypoactivation in the prefrontal cortex, such as diminished activation of the dorsal lateral prefrontal cortex (dlPFC) (Erk et al., 2010); enhanced activation of the anterior cingulate (Beauregard et al., 2006), lateral orbital-frontal cortex (Kanske et al., 2012), and right ventral medial prefrontal cortex (vmPFC) (Johnstone et al., 2007); and/or deficit in suppressing activation in limbic structures such as the amygdala and insula (Beauregard et al., 2006; Johnstone et al., 2007; Erk et al., 2010; Kanske et al., 2012), while the self-reported regulation success of depressed patients remains intact (Johnstone et al., 2007; Erk et al., 2010; Wang et al., 2014).

One explanation for the inconsistent neural findings pertaining to depression-related differences may be the diversity of emotion regulation strategies. This explanation could be evidenced by the fact of the divergence of prefrontal activations for other emotion regulation strategies, such as expression suppression (LPFC), distraction (parietal regions) and mindfulness (dlPFC/dmPFC) (Livingston et al., 2015; Morawetz et al., 2016). Even when reappraisal was concerned, different strategies of reappraisal such as reinterpretation (vlPFC) and distancing (parietal regions) recruited different prefrontal regions (Dörfel et al., 2014). Taken together, the previous literature review suggested that different emotion regulation strategies recruit both convergent and divergent activations in prefrontal regions (Morawetz et al., 2016).

Another explanation might be the confounding effects of motivation in emotion regulation. Theoretically, emotion regulation involves the pursuit of desired emotional goals in the service of hedonic or instrumental motives (Tamir, 2016). Hedonic motives include approach motivation steering toward appetitive stimuli and avoidance motivation directing away from aversive stimuli, which depend on two independent neurobiological systems—the behavioral activation system (BAS) and the behavioral inhibition system (BIS) (Corr, 2008). Previous evidence has supported the modulatory role of motivation in emotion regulation. BAS/BIS could bias higher-order cognitive control toward context-dependent regulation of emotion (Gray and Braver, 2002). Drive and fun-seeking (sub-dimensions of BAS) have demonstrated unique positive associations with adaptive ER (Tull et al., 2010). By contrast, strong BIS sensitivity and weak BAS-reward may predispose for difficulties regulating emotions, which in turn resulted in greater depression and other mental symptoms (Markarian et al., 2013).

Furthermore, hedonic motives may modulate the group differences in neural substrates of emotion regulation processes. Previous study suggested that depressed patients with higher BIS scores less recruited left ventral lateral PFC (vlPFC), a cognitive control region which was implicated in reappraisal for both groups. Depressed patients with higher BAS scores exhibited less signal change in amygdala during down regulation of their negative emotion. However, the similar relationships were not observed in healthy controls (Johnstone et al., 2007). Collectively, these results suggested that besides the motivation disposition deficits (heightened BIS levels and dampened BAS levels), the involvement of motivation in emotion regulation may differentiate between depressed vs. non-depressed individuals.

However, it remained to be tested whether BAS/BIS modulated cognitive control and emotion generation neural regions during other emotion regulation processes (e.g., up-regulation of positive/negative emotion, down-regulation of positive emotion). Typically, the participants in the reappraisal study were instructed to either increase (“enhance”) or decrease (“suppress”) the elicited emotional response. However, valence but not hedonic motives (approach/avoidance) are manipulated in such experiment context (Rottenberg, 2017). We proposed that motivation could be manipulated by distinguishing between approach-oriented (immersion) and avoidance-oriented (detachment) reappraisal. Thus in service of instrumental goals, the participants may be instructed to be psychologically distanced from the emotion stimuli to calm down (avoidant strategy), or immersed in the emotion context without approaching a solution (approach strategy) (Ayduk and Kross, 2010; Kross and Ayduk, 2011). In the experiment context, behavior is not always oriented toward the hedonic goals of momentary experience of pleasure or pain, but sometimes steering toward avoiding the positive and approaching the negative stimuli. Likewise, in daily life, behavior may be motivated toward maximizing pleasure and minimizing pain in the future (Higgins, 2012). Therefore, our first hypothesis was that approach/avoidance motivation differentially modulates reappraisal in depressed patients vs. normal healthy controls.

Due to widespread and interdependence of the neural networks of motivation and emotion regulation (Ernst and Fudge, 2009; Ernst, 2014), we mainly focused on those prefrontal-limbic regions which reliably distinguish between approach- and avoidance-oriented reappraisal (immersion/detachment). First, avoidance-oriented reappraisal (distancing) seems to recruit parietal regions which involve changing the perspective from which stimuli are understood and experienced (Ochsner et al., 2012). Approach-oriented reappraisal (immersion) selectively recruited left rostral medial prefrontal cortex (BA9/10) and posterior cingulate cortices which involve generating words that describe the emotional events (Ochsner et al., 2004). Second, ample evidence has indicated that hedonic motivates (BAS/BIS) predicted specific cognitive control abilities (Prabhakaran et al., 2011), and moderated activation in frontal cortex (e.g., MFG, dlPFC) associated with cognitive control (Spielberg et al., 2011, 2012; Bahlmann et al., 2015). More evidence also indicated that BIS modulates the amygdala/insula response (Reuter et al., 2004; Cunningham et al., 2010) and BAS correlates with ventral PFC and striatum activity in reaction to positive stimuli (appetitive pictures, monetary reward) (Beaver et al., 2006; Locke and Braver, 2008; Simon et al., 2010). Therefore, our second hypothesis was that these regions of interest (PFC, amygdala and striatum) may be differentially recruited between groups when taking covariates of BAS/BIS into consideration. The biased modulatory role of motivation underlying emotion regulation of depressed patients may not only help clarify the mechanism of emotion dysregulation of major depression, but also guide more personalized psychological intervention by addressing specific motivation deficits in MDD.

## Materials and methods

### Participants

Twelve currently drug-free, major depressed outpatients and 15 normal controls (MDD: male/female = 5/7; HC: male/female = 7/8) were recruited and evaluated by structured clinical interview for DSM-IV-TR Axis I (SCID I) (Lowe et al., 2004). The patients were screened via diagnoses from an experienced psychiatric clinician according to DSM-IV-TR. The recruited participants have had a major depressive episode, without history of neurological disease or presence of axis I psychiatric disorders, with no use of psychiatric medicine for at least 2 weeks. The healthy control group had no current or past axis I disorders and no first-degree family history of MDD, bipolar disorder, or schizophrenia. This study was approved by Ethics Committee of Third Military Medical University. The written consent form of each participant was obtained before they conducted the experiment.

### Materials

#### Emotion stimuli

Pictures of stimuli were selected from the International Affective Picture System (IAPS) (Lang et al., 2008) based on normative ratings and were matched for content of scenes and people (Table S1). Valence and arousal ratings of pictures in each session and each condition were kept homogeneous, with non-significant differences in an ANOVA (emotion × reappraisal) (*P*s > 0.05) (Wang et al., 2014). Twenty-four trials (12/positive; 12/negative) were included in the “detach/immerse” condition, and 36 trials (12/positive; 12/negative; 12/neutral) were included in the “attend” condition. Therefore, the neutral pictures were only presented under the “attend” condition. A different set of affect arousing images was selected for the practice blocks to avoid confounding effects.

#### BIS/BAS scale

We adopted a revised Chinese version of the Behavioral Inhibition System and Behavioral Activation System Scale (BBS) immediately after the scan. This scale was confirmed to be reliable and valid among Chinese populations. The Cronbach α of the total scale and the BIS, BASR, BASD, and BASF subscales were respectively 0.70, 0.59, 0.72, 0.66, and 0.55. The four-factor model of the Chinese revised version of BBS was selected because the four-factor model indicated a better model fit (AIC_two-*factor*_ < AIC_four-*factor*_, RMESA < 0.05, GFI, AGFI, IFI, CFI > 0.90) than the two-factor model (BIS, BAS) (RMESA = 0.082, GFI = 0.847, AGFI = 0.805, IFI = 0.613, CFI = 0.600, AIC = 445.620) (Li et al., 2008).

#### Beck depression inventory (BDI)

BDI is the most widely used self-rating scale which is the revised version of BDI according to the DSM-IV. BDI consists of 21 items of emotional, cognitive, motivational and somatic symptoms, which are scored from 0 (symptom not present) to 3 (symptom very intense). The BDI had a 1-week test–retest reliability of *r* = 0.93 and an internal consistency α = 0.91. Scores with 0–4 indicates normal, 5–7 mild depression, 8–15 moderate depression, and 16–63 severe depression (Beck et al., 1996).

#### Zung self-rated depression scale (SDS)

SDS consists of 20 items of psychological and somatic symptoms, which are scored from 1 (a little of the time) to 4 (most of the time). SDS has a split-half reliability of 0.73 and internal consistencies ranging from 0.68 to 0.82. Scores greater than 50 indicate mild depression, greater than 60 indicate moderate depression, and greater than 70 indicate severe depression (Zung, 1986).

#### Hamilton depression rating scale (HAMD-24)

HAMD-24 is the most widely used interview scale to measure severity of depression in an inpatient population. Scores of 0–7 are considered normal, and scores greater than or equal to 20 indicate moderately severe depression (Hamilton, 1960; Williams, 2001).

### Experimental procedure

Prior to the experiment, the participants practiced the three conditions with a different set of emotional pictures to become familiar with the task and emotion regulation strategies.

The task was performed in three consecutive blocks (“ATTEND,” “DETACH,” and “IMMERSE”). Block design was utilized to avoid potential task-switching effects that might obscure differences between regulation and passive viewing conditions (Moser et al., 2010). During the ATTEND block (as baseline condition), the subjects responded naturally without trying to change the emotional state elicited by the stimuli. During the DETACH block (avoidance-oriented reappraisal), participants were asked to interpret the situation depicted as fake or unreal, as would someone with no personal attachment to the events. During the IMMERSE block (approach-oriented reappraisal), subjects were asked to perceive each picture as real by imagining themselves or a loved one in the scene. The distinction between strategies (detach/immerse) was orthogonal within valence such that immersion was “good” for positive pictures and “bad” for negative ones, while detachment was “good” for negative pictures and “bad” for positive ones. The order of the other two blocks (DETACH/IMMERSE) was counterbalanced across participants. Within each block, the order of trials contributing to that block's 2 (ER) × 2 (emotion) design was randomized (Moser et al., 2010).

At the start of each block, a cue instruction was presented for 10 s. After a fixation period of 2 s, one of the twelve pictures used for each valence condition (positive/negative/ neutral) appeared for 8 s on the screen. Then, the participants pressed four buttons (within 4 s) with two fMRI compatible joysticks (SA-9800 E, http://www.sinorad.com/) connected to an E-prime 2.0 system (Psychology Software Tools, Sharpsburg, PA, USA), which registered their self-reported ratings of emotional intensity on a 4-point Likert scale (1 = barely not; 2 = weak; 3 = relatively strong; 4 = very strong). There was an 8-second break before the next trial to commence. The protocol for the paradigm was administered using the commercial software package E-Prime 2.0 (standard version). After scanning, the participants were asked to elaborate on the strategies used to confirm the effectiveness of emotion regulation.

### MRI data acquisition

MRI data were collected on a Siemens 3T Tim Trio MRI system (Erlangen, Germany). Sessions included an auto-align localizer, a T1-weighted MPRAGE structural image (slice thickness = 4 mm, field of view (FOV) = 240 × 240 × 240 mm^3^, matrix = 256 × 256 × 256) and three functional sessions. Functional sequence was obtained with a time repetition (TR) of 2,000 ms, a flip angle of 90°, a time echo of 30 ms, an FOV of 240 × 240 mm^2^, a matrix of 64 × 64, a slice thickness of 4 mm, and a slice interval of 0.8 mm. During scanning, visual stimuli were presented to the participants through the goggles mounted on the head coil.

### Data analysis

#### Behavioral data

The magnitude of the emotion regulation effect was measured by the change in subjective emotion ratings between the “detach/immerse” and “view” conditions for each valence of emotion. The present study was an extension of our prior study, based on the published dataset (Wang et al., 2014). We compared the BBS subscales scores between groups by performing two independent sample *t*-tests using SPSS software (Version 19, SPSS Inc., Chicago, IL, USA). To rule out the possibility that group differences in the BBS subscale scores would be partially explained by gender effects (Knyazev et al., 2004), we performed a multivariate analysis of variance (MANOVA) to test whether the motivational scores differed across the groups and/or genders. To optimize the homogeneity of the samples, outliers over 3 standard deviations away from the mean were diagnosed and excluded, and we used box-plot methods and Cook's distance to detect outliers in SPSS.

#### Functional MRI data

All functional and structural image processing and statistical analyses were conducted with SPM8 (http://www.fil.ion.ucl.ac.uk/spm/software/spm8/). The first trial of each block (attend/detach/immerse condition) was discarded to reach the magnetization equilibrium. The remaining volumes were corrected for slice timing, and then realigned to the mean volume to correct for head motion. None of the participants had head motion exceeding 3 mm translation or 3° of rotation across all volumes. Images were spatially normalized to the standard MNI space using a 12-parameter affine transformation, and smoothed by convolution with a standard 8-mm full-width at half-maximum (FWHM) isotropic Gaussian kernel. The whole-brain voxel-wise analysis based on multiple linear regression model was used. Each condition was modeled using a box-car function convolved with a canonical hemodynamic response function (HRF). The realignment parameters were also included in the models as covariates of no interest.

First, to examine the group-related differences in emotion regulation, we conducted a between-group comparison of whole-brain activations under each ER condition. Second, to test the hypothesis that motivation dispositions differentially modulated reappraisal-related brain responses in two groups, we conducted a voxel-wise analysis of covariance (ANCOVA) with group, emotion and ER as between-subject factors and BIS/BAS subscale scores as covariates. The interactive effects between group and motivation, as well as the main effect of group on emotion regulation were examined. In addition, to examine the group-related differences in motivation, we conducted a two-sample *t*-test with the BIS/BAS scores as covariates. Third, for each group and each contrast (reappraisal vs. attend), we conducted a one-sample *t*-test by entering the BIS/BAS scores as covariates of interest to identify clusters that show a linear relationship with BIS/BAS scores. Finally, we examined the correlations between BIS/BAS scores and the time courses of a priori regions of interest (ROIs) (PFC/amygdala). Those neural correlates of motivation by group interaction across ER conditions (striatum, e.g., midbrain and lentiform nucleus) were also examined.

##### Definition of ROIs

Based on previous neuroimaging studies on emotion regulation (Beauregard et al., 2006; Johnstone et al., 2007; Abler et al., 2010; Erk et al., 2010; Kanske et al., 2012), the following ROI criteria were identified for further analysis: bilateral dlPFC (BA9,46) and bilateral vmPFC (BA10,11,32,25). To produce the ROIs, we used masks derived from WFU PickAtlas software (version 3.0; ANSIR Laboratory, WFU School of Medicine, Winston-Salem, North Carolina) with a threshold of *p* < 0.05 and an extent threshold of 5 voxels. ROI time courses were extracted within anatomically pre-defined ROIs by generating the first eigenvariate of 8 mm around the peak voxels using the MATLAB package REX (Response Exploration) (Duff et al., 2007). A corrected threshold of *P* < 0.01 (two-tailed) for multiple comparison was derived from a combined threshold of *P* < 0.05 for each voxel and a cluster size of greater than 54 voxels using the AlphaSim program embedded in the REST software program (http://www.restfmri.net/forum/REST_V1.5). The parameters were as follows: single voxel *p* < 0.01, 1,000 iterations, FWHM = 4 mm, and a gray matter mask. We adjusted for multiple comparisons between Pearson correlations using Bonferroni correction, with a corrected threshold of *P* < 0.003 (= 0.05/15).

## Results

### Group differences in demographic and clinical variables

The two groups were matched for age (average age; MDD: 29.50 ± 8.46 SD; HC: 25.80 ± 5.89 SD) and education (average years; MDD: 14.00 ± 3.77; HC: 14.80 ± 2.83) (*P* > 0.05). The patient and control groups did not differ in terms of age, education level or gender ratio (*P*s > 0.05). Significant differences were found in BDI and SDS scores between the two groups (*Ps* < 0.05). Average scores of BASD and BASR for the patient group were lower than those for the control group (*P* = 0.036 and 0.002), and the BIS score for the patient group was higher than that of the control group (*P* = 0.049). No significant group difference was detected with respect to BASF scores (*P* > 0.05) (Table 1).

**Table 1:** Group comparison of demographic, clinical, and neuropsychological variables.

**Variables**	**HC (*n* = 15)**	**MDD (*n* = 12)**	***P-*value**
	**Mean ± SD**	**Mean ± SD**	
Age	25.80 ± 5.89	29.50 ± 8.46	0.088
Education (years)	14.80 ± 2.83	14.00 ± 3.77	0.094
Gender ratio (M: F)	7/8	5/7	0.841
BDI	4.27 ± 4.23	26.17 ± 12.65	<0.001^**^
SDS	36.54 ± 5.74	64.08 ± 12.60	<0.001^**^
HAMD-24	NA	25.17 ± 5.18	
Number of previous episodes	NA	1 in 9/12 patients 2 in 2/12 patients 3 in 1/12 patients	
BIS	14.87 ± 2.13	16.67 ± 2.39	0.049^*^
BASD	12.53 ± 2.59	10.42 ± 2.31	0.036^*^
BASR	14.53 ± 1.19	12.50 ± 1.93	0.002^**^
BASF	14.80 ± 2.18	14.08 ± 1.62	0.352

* *P < 0.05*.

** *P < 0.01; NA, not applicable*.

The results showed that BIS, BASD and BASR differed between groups. Multivariate analysis of variance (MANOVA) revealed that the main effects of gender (Wilks' Lambda *F* = 1.297, *P* = 0.305, η^2^ = 0.305) and gender-by-group interaction were not statistically significant (Wilks' Lambda *F* = 1.160, *P* = 0.358, η^2^ = 0.188), thus ruling out the possibility that group differences in motivation dispositions would be partially explained by gender effects (Table 2).

**Table 2:** Gender effects on motivation disposition profiles.

**Variables**	**Group**	**Male (x ± SD)**	**Female (x ± SD)**	**Levene' s test**	**Box's *M*-test**	**Mean difference**	**Std. error**	***P-*value**
				***P-*value**				
BIS	MDD	17.00 ± 2.23	16.43 ± 2.64	0.197	*p* = 0.28^a^	−0.26	0.90	0.775
	HC	14.29 ± 1.11	15.38 ± 2.72					
BASD	MDD	11.60 ± 2.07	9.57 ± 2.23	0.616		1.85	0.93	0.058
	HC	13.43 ± 1.99	11.75 ± 2.92					
BASR	MDD	12.40 ± 1.34	12.57 ± 2.37	0.293		0.08	0.63	0.896
	HC	14.71 ± 1.38	14.38 ± 1.06					
BASF	MDD	15.40 ± 0.89	13.14 ± 1.35	0.014^*^		1.05	0.73	0.165
	HC	14.71 ± 2.06	14.88 ± 2.42					

* *P < 0.05*.

a *Box's M test confirmed the equivalence of covariance matrices across levels of the independent variables*.

#### Relationship between BIS/BAS scores and depressive severity

For the MDD group, BIS scores were positively correlated with BDI (*r* = 0.860, *P* < 0.001, *n* = 12). No statistically significant correlations between BIS scores and depressive symptoms were found for the control group (*Ps* > 0.05). No statistically significant correlations between BAS scores and depressive severity for both groups.

#### Relationship between BIS/BAS scores and emotion (regulation)

The emotion regulation effects were comparable between the two groups, which result was reported in the previous study (Wang et al., 2014). The correlations between BIS/BAS scores and emotion responding/regulation effects were analyzed. Positive association was observed in the control group between BAS-drive and negative affect (attend/negative vs. attend/neutral) (*r* = 0.614, *P* = 0.024, *n* = 13). However, this association was not observed in the MDD group (*P* > 0.05). The correlations between BIS/BAS and positive emotion, as well as between BIS/BAS and the emotion regulation effects (positive/detach; negative/detach; positive/immerse; negative/immerse) were not significant for both groups (*Ps* > 0.05).

### Functional MRI data

#### Group differences in neural activation under each emotion regulation condition

For “detach-attend” contrasts of positive and negative stimuli, lower activations in the posterior cingulate (PCC) and para-hippocampal gyrus (PHG) and greater activations in the middle and superior temporal gyrus (MTG, STG) were found in depressed patients. For “immerse-attend” contrasts of positive and negative stimuli, similar results were observed in depressed patients (Table 3). Collectively, these results demonstrated that weaker PCC/PHG and stronger MTG/STG activations could be generalized across ER conditions for the MDD group.

**Table 3:** Group differences in contrasts of “reappraisal” vs. “attend” of emotion.

**Region of activation**	**Side**	**BA**	**MNI Coordinates**			***Z* score**
			***x***	***y***	***z***	
**A. POSITIVE(DETACH-ATTEND)**						
**MDD<control**						
Posterior cingulate	R	30	22	−64	10	2.33
**MDD > control**						
Middle temporal gyrus	R	19	42	−60	18	3.02
Superior temporal gyrus	L	41	−42	−36	4	2.42
**B. NEGATIVE(DETACH-ATTEND)**						
**MDD<control**						
Posterior cingulate	L	30	−22	−62	8	3.06
Parahippocampal gyrus	L	19	−26	−50	0	2.72
Posterior cingulate	R	30	20	−66	16	2.52
Parahippocampal gyrus	R	30	32	−52	6	2.51
**MDD > control**						
Middle temporal gyrus	L	22	−52	−46	2	2.13
**C. POSITIVE(IMMERSE-ATTEND)**						
**MDD<control**						
Parahippocampal gyrus	R	36	32	−40	−10	2.00
Lingual gyrus	R	18	14	−82	6	1.70
**MDD > control**						
Superior temporal gyrus	L	41	−54	−28	18	3.06
Insula	L	13	−50	−6	12	2.79
Inferior parietal lobule	R	40	56	−28	22	2.07
Caudate	R		20	2	24	2.31
**D. NEGATIVE(IMMERSE-ATTEND)**						
**MDD<control**						
Parahippocampal gyrus	L	36	−26	−44	−10	2.71
Parahippocampal gyrus	R	37	36	−44	−14	1.72
Lingual gyrus	R	19	22	−62	−2	2.00
**MDD > control**						
Middle temporal gyrus	R		50	−38	−6	2.38
Superior temporal gyrus	R	21	54	−26	−8	1.98

*All clusters were thresholded at P < 0.05 and AlphaSim-corrected with an extent of at least 54 voxels*.

#### Group effects on motivation-related brain responses underlying emotion regulation

ANCOVA analysis revealed the left midbrain activation (MNI coordinates: −6, −32, 0, *Z* = 2.82, cluster size: 3,967) underlying the interactive effect between group and motivation. As for the group differences in motivation-related neural substrates during reappraisal, in addition to those regions with group differences (PCC, PHG, STG, MTG) without adjusting for BIS/BAS covariates under each condition (Table 3), additional regions such as the bilateral inferior frontal gyrus (IFG, BA45) and lentiform nucleus were also observed (Table 4). These results suggested that the IFG and lentiform nucleus may play an essential role in approach/avoidance motivation which differentiated the MDD group from the HC group. Specifically, across the “detach” and “immerse” conditions for the depressed patients, lower IFG (BA45) activation was modulated by BASD, BASR, and BIS scores; lower right lentiform nucleus activation was modulated by BASD and BIS scores; and greater left lentiform nucleus activation was modulated by BASF scores (Table 4).

**Table 4:** Motivation effects on group-dependent brain activities during reappraisal.

**Region of activation**	**Side**	**BA**	**MNI coordinates**			**Cluster size**	***Z* score**
			***x***	***y***	***z***		
**A. BASD**							
**Control > MDD**							
Inferior frontal gyrus	L	45	−28	34	−4		2.16
Posterior cingulate	R	23	12	−34	18		2.56
Posterior cingulate	L	29	−12	−44	18	695	2.39
Lentiform nucleus	R		22	−20	2	87	2.28
Parahippocampal gyrus	L	36	−28	−34	−10	196	2.1
Middle temporal gyrus	L	39	−40	−66	20	105	2.01
Superior temporal gyrus	L	39	−48	−54	16		1.82
**B. BASR**							
**Control > MDD**							
Inferior frontal gyrus	R	45	46	22	10	55	2.02
Parahippocampal gyrus	R	19	42	−46	−8		2.93
Posterior cingulate	R	30	28	−70	10	361	
Anterior cingulate	R	32	14	40	14	72	
**C. BASF**							
**Control > MDD**							
Posterior cingulate	L	29	0	−36	−20	64	2.25
Superior temporal gyrus	L	13	−50	−46	24		1.82
**MDD > Control**							
Lentiform nucleus	L		−28	−8	−2	93	2.12
Midbrain	L		−2	−20	−4	52	2.05
Lentiform nucleus	R		30	−8	2	42	1.82
**D. BIS**							
**Control > MDD**							
Inferior frontal gyrus	L	45	−36	34	218		2.29
Lentiform nucleus	R		28	8	4	939	2.32

*The group-by-motivation interaction identified regions where BIS/BAS scores modulated brain responses differently between the depressed patients and the control group regardless of reappraisal conditions. All clusters were thresholded at P < 0.05 and AlphaSim-corrected with an extent of at least 54 voxels*.

Next, we examined the neural substrates underlying the main effect of motivation (IFG, lentiform nucleus) as well as the interactive effect between motivation and group (midbrain) under each ER condition. (1) **IFG**. Comparison of motivation-related neural correlates between groups under each ER condition did not yield significant IFG activation. For each group, no IFG activation was found under each ER condition. (2) **Lentiform nucleus**. Normal individuals exhibited more activation in lentiform nucleus under positive/detach and negative/detach conditions, which was modulated by BAS (BASR/BASF). Depressed patients demonstrated more activation in lentiform nucleus under positive/immerse and negative/immerse conditions, which was modulated by BIS. (3) **Midbrain**. Normal individuals exhibited more activation in midbrain under positive/detach and negative/detach conditions, which was modulated by BASR. Depressed patients demonstrated more activation in midbrain under positive/immerse and negative/immerse conditions, which was modulated by BASF and BASF/BIS respectively (Table S2).

#### Motivation dispositions modulate neural responses ER-related regions of interest in each group

##### PFC

(1) For normal individuals, bilateral dlPFC (BA9) and vmPFC (BA10) were activated under the *Positive (detach-attend)* and *Negative (detach-attend)* conditions, which were modulated by BIS and BASD respectively. Control subjects also exhibited increased ventral lateral PFC (vlPFC) (BA47) activation modulated by BASR under the *Negative (detach-attend)* condition. (2) For depressed patients, left dlPFC (BA9) was activated under the *Negative (immerse-attend)* condition which was modulated by BASD (Table 5).

**Table 5:** BIS/BAS modulated regions during reappraisal in healthy and depressed groups.

**Region of activation**	**Side**	**BA**	**MNI Coordinates**			**Cluster size**	***Z* score**
			***x***	***y***	***z***		
**A. POSITIVE (DETACH-ATTEND)**							
**Control**							
[BIS]							
Inferior frontal gyrus	R	47	46	22	−28	188	2.86
Middle frontal gyrus	R	10	32	54	−12	56	2.11
[BASR]							
Inferior frontal gyrus	R	47	26	32	−14		3.56
Midbrain	R		4	−12	−20		3.44
[BASD]							
Midbrain	R		0	−30	−12	104	2.04
**MDD**							
[BIS]							
Posterior cingulate	R	23	14	−58	16	1460	2.93
Middle frontal gyrus	R	9	42	22	26		2.02
**B. NEGATIVE (DETACH-ATTEND)**							
**Control**							
[BASD]							
Medial frontal gyrus	R	10	6	56	−4	107	2.90
Anterior cingulate	L	32	−22	36	14	86	2.74
Midbrain	R		4	−24	−16	96	2.66
[BASR]							
Hippocampus	L		−26	−46	8	385	2.45
Medial frontal gyrus	R	10	14	54	2	74	2.36
**MDD**							
[BASD]							
Middle frontal gyrus	L	9	−40	20	20		2.59
Middle frontal gyrus	R	9	42	28	22		2.32
[BASR]							
Midbrain	L		0	−18	−6		2.23
Posterior cingulate	L	30	0	−62	14	88	2.20
**C. POSITIVE (IMMERSE-ATTEND)**							
**Control**							
[BASD]							
Midbrain	R		4	−24	−16	96	2.66
[BASR]							
Midbrain	R		6	−24	−14		2.53
Middle frontal gyrus	R	46	42	34	8	113	2.42
Cingulate gyrus	L	24	−6	4	24	110	2.36
Amygdala	L		−26	−4	−20	59	2.25
[BASF]							
Middle frontal gyrus	R	10	40	40	−2	165	3.51
Medial frontal gyrus	R	10	22	52	2	89	2.46
Medial frontal gyrus	R	10	18	40	−18	84	2.41
**MDD**							
[BASD]							
Cingulate gyrus	R	24	2	−2	42	659	3.03
[BASF]							
Amygdala	R		32	−12	−18		2.02
[BIS]							
Medial frontal gyrus	L	9	−20	44	18	160	2.36
**D. NEGATIVE (IMMERSE-ATTEND)**							
**Control**							
[BIS]							
Midbrain	L		−4	−8	−12	58	2.76
**MDD**							
[BASD]							
Middle frontal gyrus	L	9	−42	18	28	11,221	4.39
Lentiform nucleus	L		−26	−4	8		4.05
Inferior parietal lobule	R	40	44	−24	44	532	3.14

*A one-sample t-test for each group and for each condition was performed, and BIS/BAS scores were entered as covariates of interest, which yielded whole-brain activation. All clusters were thresholded at P < 0.05 and AlphaSim-corrected with an extent of at least 54 voxels*.

##### Midbrain and lentiform nucleus

(1) For normal individuals, midbrain was activated (a) under the *Positive (detach-attend), Positive (immerse-attend)* and *Negative (detach-attend)* conditions, which was modulated by BASD/BASR, and (b) under the *Negative (immerse-attend)* condition which was modulated by BIS. (2) For depressed patients, midbrain was activated under the *Negative (detach-attend)* condition, which was modulated by BASR. Additionally, depressed patients demonstrated enhanced lentiform nucleus activation under the *Negative (immerse-attend)* condition, which was modulated by BASD (Table 5).

##### Amygdala

Under the *Positive* (*immerse-attend)* condition, depressed patients exhibited heightened activations in the right amygdala modulated by BASF (Table 5). The result complemented with our previous observation of enhanced right amygdala activation in this contrast (Wang et al., 2014). Furthermore, the Pearson correlation between the self-reported emotion enhancement effect and the neuroimaging signal change in the right amygdala under this condition was significant (*r* = 0.594, *P* = 0.042, *n* = 12) for the MDD group.

To obtain complementary evidence, we also computed the intensity of peak activation derived from functional ROIs under each condition, as well as the correlations between brain activations and BIS/BAS scores. Only significant correlations were reported here. (1) For depressed patients, right dlPFC (BA9; peak MNI coordinates: *x* = 42, *y* = 22, *z* = 26) activation was negatively modulated by BIS scores when they detached from positive emotional stimuli, and when we entered BDI scores as a predictor into the GLM, BIS, and BDI scores jointly predicted right dlPFC activation, with additional variance derived from depressive symptoms (from 31.3% to 59.6%) (Table 6). (2) For healthy controls, right vmPFC (BA10; peak MNI coordinates: *x* = 14, *y* = 54, *z* = 2) activation was positively modulated by BASR scores in healthy controls when they detached from negative emotional stimuli (Table 6).

**Table 6:** Motivational dispositions Predict Reappraisal-related Brain Activity in functional ROIs.

**Group**	**Contrast**	**Brain region**	**BBS Subscales**	**Significance**	**Standard coefficients**	**Adjusted *R*^2^ (*P*)**
MDD	Positive (detach-attend)	R_dlPFC	BIS	0.045^**^	−0.789	0.313 (0.164)
		R_dlPFC	BIS	0.002^**^	1.561	0.596 (0.007)
			BDI	0.003^**^	−1.532	
HC	Negative (detach-attend)	R_vmPFC	BASR	0.031^**^	0.713	0.434 (0.043)

*BIS/BAS scores were entered into a generalized linear regression model to predict reappraisal-related brain activation. Functional ROI time courses (beta values) under each condition in each group were generated by extracting the first eigenvariate of 8 mm around the peak voxels of respective clusters with no scaling, using a Matlab package REX (Response Exploration). BBS, BIS/BAS scale; L, left hemisphere; R, right hemisphere*.

** *P < 0.01*.

**Figure 1:**
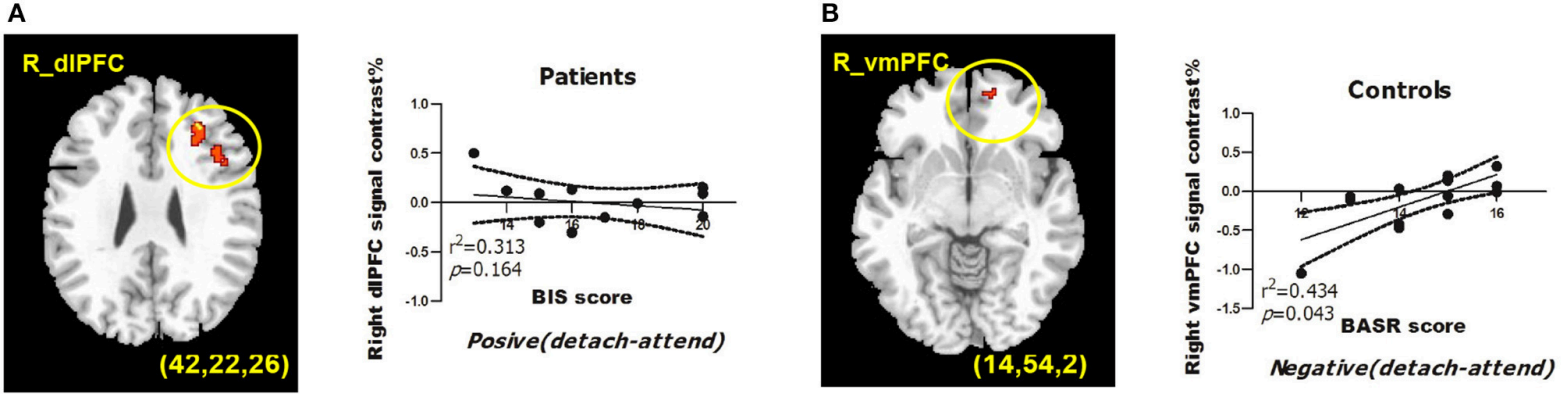
BIS/BAS measures predict BOLD signals from regions of interests during reappraisal of emotions. **(A)** BIS scores predict right dlPFC activation under detach-positive condition for depressed patients. **(B)** BASR scores predict right vmPFC activation under detach-negative condition for healthy controls. MDD, depressed patient group; HC, healthy control group. The correlation coefficients and significance were indicated below each panel. All clusters survived the voxel-wise whole-brain analysis Alpha-sim corrected at *p* < 0.05 with an extent of at least 54 voxels.

For depressed patients, BIS scores negatively predicted right dlPFC (BA9; peak MNI coordinates: *x* = 42, *y* = 22, *z* = 26) activation when they detached from positive emotional stimuli. For healthy controls, BASR scores positively predicted right vmPFC (BA10; peak MNI coordinates: *x* = 14, *y* = 54, *z* = 2) activation when they detached from negative emotional stimuli (Figure 1).

## Discussion

The present study demonstrated the modulatory role of motivational dispositions during cognitive regulation of emotion, and the dysfunctional motivated regulation of emotion for major depressive disorder. Behaviorally, our results confirmed the approach and avoidance motivation deficits of MDD, with lower levels of BAS-reward responsiveness and BAS-drive and higher levels of BIS. Furthermore, BIS levels were related to the severity of depressive symptoms of MDD group. These results support the claim of BAS and BIS sensitivities as stable markers of mood disorders (Henriques and Davidson, 2000; Fletcher et al., 2013; Quilty et al., 2014). Specifically, higher BIS sensitivity may increase the avoidance goals and behaviors and amplify affective reactions to negative events (Gable et al., 2000) and is responsible for the excessive negative emotion observed in MDD. In contrast, lower BAS functioning may be associated with approach deficits which limit the access to positive emotion and rewarding experiences (Trew, 2011) and in turn lead to sustained negative affect. Consistent with this assumption, lower BAS-drive are positively related to greater negative affect (compared to viewing neutral stimuli) in the control group. However, we did not find any other correlation between the BAS/BIS and positive/negative affect. Moreover, we did not find any significant association between motivation and ER effects, which was also not observed in the relevant study which examined the modulatory role of motivation in emotion regulation (Johnstone et al., 2007). Therefore, we further examined the modulatory role of motivation in the neural correlates of emotion regulation. Before that, abnormal neural substrates of emotion regulation were examined in depressed patients.

### Abnormal neural correlates of emotion regulation in depressed patients

Although stronger MTG and STG were activated during emotion regulation for the MDD group, MTG and STG were less modulated by BAS across ER conditions. The hypoactivation of MTG for clinical depression is consistently activated in fMRI cognitive reappraisal studies (Pico-Perez et al., 2017). Greater MTG activation of depressed patients may represent more resources devoted to lexical representation and retrieval (Huang et al., 2012), and processing emotionally laden negative stimuli (Paquette et al., 2003; Jessen and Kotz, 2015). STG is involved in reinterpretation of emotion stimuli (Pico-Perez et al., 2017). Thus stronger STG activation observed in depressed patients may reflect novelty detection (Dominguez-Borras et al., 2009) to visual stimuli with medium to high arousal (Mather et al., 2006). Therefore, enhanced MTG/STG activations less modulated by BAS-drive for the MDD group may reflect dysfunction in the goal-directed system.

Moreover, less PHG and PCC were activated during emotion regulation for the MDD group, while PHG and PCC were less modulated by BAS across ER conditions. The PHG and PCC were critical to emotion regulation. The PHG is implicated in the early appraisal and encoding of emotional significance during regulation of behavioral responses (Almeida et al., 2009). The PHG showed decreased activation during down- and increased activation levels during upregulation of emotion (Frank et al., 2014). Our result was contradictory with previous observation of hyperactivation of the PHG during positive reappraisal (actively make a negative picture more positive) in depressed patients (Sheline et al., 2009). Hypoactivation of the PCC has been reliably reported in the cognitive reappraisal of depressed patients (Pico-Perez et al., 2017). PCC has strong reciprocal connections with parahippocampal cortices and plays an important role in successful retrieval of autobiographical memories (Maddock et al., 2001), which is critical to the deployment of successful self-focused reappraisal strategies. Therefore, the lack of recruitment of PCC may be related to deficits in approach motivation.

Overall, the approach motivation may be differentially involved in those neural regions implicated in different stages of emotion processing, which leads to biased early stage salience processing, semantic processing and self-relevant memory retrieval.

### Biased modulatory role of motivation underlying the neural correlates of emotion regulation

Both BAS and BIS sensitivity modulated the IFG (vlPFC, BA45) and lentiform nucleus differentially between groups during emotion regulation. Therefore, these two regions may be key regions implicated in the integration of motivation and emotion regulation.

#### Prefrontal regions

IFG hypoactivation of MDD group was observed for motivation-related neural correlates across ER conditions. First, our result supported the role of IFG in reappraisal, which region becomes more effective at supporting reappraisal with age (Belden et al., 2015). Abnormal function of IFG in reappraisal may exhibit in two ways: (1) less IFG activation was found in children with MDD history (Belden et al., 2015), which is aligned with our result. (2) Although comparable IFG activation was found in adult MDD patients, this region is not mediated by vmPFC to down regulate the amygdala activation (Johnstone et al., 2007). Second, our result did not yield the main effect of motivation (either across the ER conditions or under each ER condition) or the interactive effect between motivation and group on IFG activation. In contrast, our previous study support the role of the IFG as the interactive region of reappraisal and group (Wang et al., 2014).

Despite a higher level of avoidance motivation and its contradictory effect on right dlPFC activation, depressed patients still showed heightened right dlPFC activation during decreasing positive emotion. Our results echoed with the role of dlPFC as critical for distancing from emotions (Hutcherson et al., 2012) and modulating the vmPFC representation of the values assigned to stimuli. Therefore, the contradictory effect of avoidance motivation (increased BIS level and its negative correlation with dlPFC activation) and exaggerated activation in right dlPFC may explain the comparable self-reported ER effects between the two groups.

Heightened right vmPFC activation could partially be explained by greater appetitive motivation when healthy controls are detached from negative emotions. Previous evidence indicates the role of vmPFC in encoding emotional value during the experience and regulation of both positive and negative emotional stimuli (Winecoff et al., 2013) Heightened right vlPFC activation could partially be explained by biased approach and avoidance motivation when healthy controls are detached from positive emotions. The vlPFC plays an essential role in both increasing and decreasing emotion (Dörfel et al., 2014; Tupak et al., 2014). Our results extended previous findings that depressed patients with higher BIS tended to recruit the vlPFC to a less extent while decreasing negative emotion (Johnstone et al., 2007). Collectively, due to the evidence that prefrontally mediated cognitive control can either inhibit or augment reactions to achieve successful goal-directed behavior (Eippert et al., 2007), the altered prefrontal emotion regulatory network (dlPFC/vmPFC/vlPFC) in depressed patients demonstrated ineffective top-down modulation of emotion, as well as impaired modulatory role of approach/avoidance motivation in emotion regulation.

#### Midbrain and lentiform nucleus

Striatal regions were observed when examining the neural substrates underlying the main effect of motivation (lentiform nucleus) and the interactive effect between motivation and group (midbrain). (1) Lentiform nucleus was differentially involved in emotion regulation process between HC and MDD group. Specifically, normal individuals recruited lentiform nucleus during the avoidance-oriented reappraisal which was modulated by BAS (BASR/BASF), while depressed patients recruited this region during approach-oriented reappraisal, which was modulated by BIS. Lentiform nucleus, part of the dorsal striatum, comprised of the globus pallidus and the putamen. Lentiform nucleus is involved in appetitive motivation and cognitive flexibility (Aarts et al., 2011; Fuentes-Claramonte et al., 2015), which function is intact in normal individuals even when they are required to be emotionally detached from the stimuli. In contrast, abnormal brain metabolism and gray matter volume of lentiform nucleus has been reported in MDD patients (Du et al., 2014; Su et al., 2014). Furthermore, heightened avoidance motivation of the MDD patients may hinder the effort to approach the stimuli and amplify the emotion responding. Accordingly, the hyperactivation of lentiform nucleus compared to normal controls during immersion was comprehensible because the lentiform nucleus is activated when MDD patients upregulated their negative emotion but not positive emotion. (2) The ventral tegmental area (VTA) which is the component of midbrain, play a role in receiving rewarding/aversive signals with motivation salience, and releasing dopamine into the ventral striatum, the amygdala and the prefrontal cortex.

#### Amygdala

Under the immerse/positive condition, greater activation in the right amygdala was found in MDD patients, which was modulated by BAS-fun seeking. The right amygdala was activated when the individual was immersed in positive emotion (Wang et al., 2014), and the signal change of right amygdala reflected the regulation effect of positive emotion. Therefore, depressed patients might maintain relatively intact hedonic motivation (comparable BASF levels) and experiences (amygdala activation) of appetitive stimuli. This result extended previous evidence that patients with higher BAS failed to decrease amygdala activation (when down-regulating emotion) (Johnstone et al., 2007).

Collectively, these results suggest that aberrant motivational disposition is implicated in the emotion dysregulation model of depression. The current study has a few limitations. *First*, because of the small sample size, caution should be taken in drawing conclusions from the analyses of this sample. However, the agreement between the behavioral and neural patterns observed in this study and those reported in previous studies justify applying the results of this study to future research. *Second*, because the present study is correlational, a follow-up study is required to manipulate the approach/avoidance motivation underlying the neural substrates of emotion regulation. Nonetheless, the clinical implications of this study merit future exploration. The relationship between individual motivation disposition and emotion dysregulation of depressed patients may guide more personalized cognitive behavioral therapy (CBT) by addressing specific motivation deficits in MDD.

## Author contributions

XW conducted the experiment, analyzed the data and drafted the manuscript; QD revised the manuscript; XZ interpreted the data; BJ conducted the fMRI scanning; ZF designed the experiment.

### Conflict of interest statement

The authors declare that the research was conducted in the absence of any commercial or financial relationships that could be construed as a potential conflict of interest.

## Abbreviations

MDD: Major depressive disorder
BIS/BAS Scale: BBS, Behavioral Inhibition System and Behavioral Activation System Scale
PCC: Posterior cingulate cortex
PHG: Para-hippocampal Gyrus
MTG: Middle Temporal Gyrus
STG: Superior Temporal Gyrus
ER: Emotion Regulation
IFG: Inferior frontal gyrus
vmPFC: Ventral medial frontal cortex
vlPFC: Ventral lateral frontal cortex
MCCE: Model of the cognitive control of emotion
dlPFC: Dorsal lateral prefrontal cortex.
