# Supplementary material for: The Role of Motivation in Cognitive Reappraisal for Depressed Patients
Source: Front Hum Neurosci. 2017 Oct 31;11:516. doi: 10.3389/fnhum.2017.00516 (PMC5671608; doi:10.3389/fnhum.2017.00516)
Supplement: Supplementary file 1 [file Table1.DOC]

**Table 1 The characteristics of selected IAPS pictures under each reappraisal condition**

| **set** | **IAPS** | **valmn** | **valsd** | **arsmn** | **arssd** | **domlmn** | **domlsd** | **affect** | **block** | **instructions** |
| --- | --- | --- | --- | --- | --- | --- | --- | --- | --- | --- |
| NeutBaby | 2250 | 6.64 | 2.26 | 4.19 | 2.28 | 6.85 | 2.28 | 1 | 1 | attend |
| Mother | 2311 | 7.54 | 1.37 | 4.42 | 2.28 | 6.16 | 1.79 | 1 | 1 | immerse |
| Children | 2345 | 7.41 | 1.72 | 5.42 | 2.47 | 6.51 | 2.07 | 1 | 1 | detach |
| Child | 2655 | 6.88 | 2.09 | 4.57 | 2.19 | 6.14 | 2.33 | 1 | 1 | attend |
| Romance | 4641 | 7.2 | 1.59 | 5.43 | 2.1 | 6.01 | 1.88 | 1 | 1 | immerse |
| Waterfal | 5260 | 7.34 | 1.74 | 5.71 | 2.53 | 4.54 | 2.56 | 1 | 1 | detach |
| Nature | 5270 | 7.26 | 1.57 | 5.49 | 2.54 | 4.28 | 2.56 | 1 | 1 | attend |
| Liftoff | 5450 | 7.01 | 1.6 | 5.84 | 2.4 | 4.98 | 2.69 | 1 | 1 | immerse |
| Astronau | 5460 | 7.33 | 1.51 | 5.87 | 2.5 | 4.99 | 2.59 | 1 | 1 | detach |
| Astronau | 5470 | 7.35 | 1.62 | 6.02 | 2.26 | 4.96 | 2.47 | 1 | 1 | attend |
| SkyDiver | 5621 | 7.57 | 1.42 | 6.99 | 1.95 | 5.81 | 2.38 | 1 | 1 | immerse |
| Mountain | 5628 | 6.51 | 1.95 | 5.46 | 2.09 | 6.28 | 2.33 | 1 | 1 | detach |
| Hiker | 5629 | 7.03 | 1.55 | 6.55 | 2.11 | 5.68 | 2.55 | 1 | 2 | attend |
| Firework | 5910 | 7.8 | 1.23 | 5.59 | 2.55 | 5.56 | 2.37 | 1 | 2 | immerse |
| Brownie | 7200 | 7.63 | 1.74 | 4.87 | 2.59 | 6.9 | 2.59 | 1 | 2 | detach |
| IceCream | 7270 | 7.53 | 1.73 | 5.76 | 2.21 | 5.88 | 2.4 | 1 | 2 | attend |
| Desert | 7580 | 7.51 | 1.6 | 4.59 | 2.72 | 5.12 | 2.56 | 1 | 2 | immerse |
| Skier | 8030 | 7.33 | 1.76 | 7.35 | 2.02 | 4.7 | 2.66 | 1 | 2 | detach |
| Skier | 8031 | 6.76 | 1.39 | 5.58 | 2.24 | 6.36 | 1.66 | 1 | 2 | attend |
| IceSkate | 8032 | 6.38 | 1.57 | 4.19 | 2.08 | 6.1 | 1.64 | 1 | 2 | immerse |
| Diver | 8040 | 6.64 | 1.56 | 5.61 | 2.01 | 5.31 | 2.32 | 1 | 2 | detach |
| Sailing | 8080 | 7.73 | 1.34 | 6.65 | 2.2 | 5.91 | 2.1 | 1 | 2 | attend |
| Athlete | 8120 | 7.09 | 1.36 | 4.85 | 2.13 | 6.23 | 1.61 | 1 | 2 | immerse |
| CliffDiv | 8180 | 7.12 | 1.88 | 6.59 | 2.12 | 4.97 | 2.52 | 1 | 2 | detach |
| Motorcyc | 8260 | 6.18 | 1.8 | 5.85 | 2.18 | 5.29 | 1.96 | 1 | 3 | attend |
| Diver | 8280 | 6.38 | 1.46 | 5.05 | 2.18 | 5.85 | 1.95 | 1 | 3 | immerse |
| Pilot | 8300 | 7.02 | 1.6 | 6.14 | 2.21 | 5.31 | 2.31 | 1 | 3 | detach |
| CarRacer | 8320 | 6.24 | 1.78 | 4.27 | 2.21 | 5.51 | 1.79 | 1 | 3 | attend |
| Winner | 8330 | 6.65 | 1.39 | 4.06 | 2.28 | 5.56 | 1.59 | 1 | 3 | immerse |
| Rafting | 8370 | 7.77 | 1.29 | 6.73 | 2.24 | 5.37 | 2.02 | 1 | 3 | detach |
| RollerCo | 8490 | 7.2 | 2.35 | 6.68 | 1.97 | 5.37 | 2.46 | 1 | 3 | attend |
| Gold | 8500 | 6.96 | 1.64 | 5.6 | 2.4 | 5.87 | 2.14 | 1 | 3 | immerse |
| Romance | 4614 | 7.15 | 1.44 | 4.67 | 2.47 | 6.62 | 1.84 | 1 | 3 | detach |
| Mountain | 5820 | 7.33 | 1.73 | 4.61 | 2.59 | 5.69 | 2.17 | 1 | 3 | attend |
| Pizza | 7350 | 7.1 | 1.98 | 4.97 | 2.44 | 6.72 | 2.12 | 1 | 3 | immerse |
| SportCar | 8510 | 7.32 | 1.72 | 4.93 | 2.56 | 6.54 | 2.26 | 1 | 3 | detach |
| Grieving | 2141 | 2.44 | 1.64 | 5 | 2.03 | 3.92 | 2.14 | 2 | 1 | attend |
| CryingBo | 2900 | 2.45 | 1.42 | 5.09 | 2.15 | 3.64 | 1.7 | 2 | 1 | immerse |
| Tumor | 3261 | 1.82 | 1.34 | 5.75 | 2.64 | 3.57 | 2.38 | 2 | 1 | detach |
| Gang | 6242 | 2.69 | 1.59 | 5.43 | 2.36 | 3.49 | 2.19 | 2 | 1 | attend |
| Suicide | 6570 | 2.19 | 1.72 | 6.24 | 2.16 | 4.03 | 2.52 | 2 | 1 | immerse |
| Needles | 9007 | 2.49 | 1.41 | 5.03 | 2.32 | 4.18 | 2.27 | 2 | 1 | detach |
| Shark | 1930 | 3.79 | 1.92 | 6.42 | 2.07 | 3.19 | 2.15 | 2 | 1 | attend |
| Cigarett | 9830 | 2.54 | 1.75 | 4.86 | 2.63 | 4.96 | 2.38 | 2 | 1 | immerse |
| Toddler | 2095 | 1.79 | 1.18 | 5.25 | 2.34 | . | . | 2 | 1 | detach |
| SadChild | 2703 | 1.91 | 1.26 | 5.78 | 2.25 | . | . | 2 | 1 | attend |
| SadChild | 2800 | 1.78 | 1.14 | 5.49 | 2.11 | 3.4 | 2.32 | 2 | 1 | immerse |
| Gun | 2811 | 2.17 | 1.38 | 6.9 | 2.22 | . | . | 2 | 1 | detach |
| Accident | 3015 | 1.52 | 0.95 | 5.9 | 2.82 | 2.84 | 2.13 | 2 | 2 | attend |
| Cigarett | 9830 | 2.54 | 1.75 | 4.86 | 2.63 | 4.96 | 2.38 | 2 | 2 | immerse |
| Injectio | 9594 | 3.76 | 1.7 | 5.17 | 2.17 | 4.43 | 2.25 | 2 | 2 | detach |
| BurnVict | 3102 | 1.4 | 1.14 | 6.58 | 2.69 | 2.18 | 1.9 | 2 | 2 | attend |
| Mutilati | 3150 | 2.26 | 1.57 | 6.55 | 2.2 | 3.39 | 2.15 | 2 | 2 | immerse |
| Attack | 6370 | 2.7 | 1.52 | 6.44 | 2.19 | 3 | 1.87 | 2 | 2 | detach |
| DeadTige | 6415 | 2.21 | 1.51 | 6.2 | 2.31 | . | . | 2 | 2 | attend |
| Starving | 9040 | 1.67 | 1.07 | 5.82 | 2.15 | 3.1 | 2 | 2 | 2 | immerse |
| PlaneCra | 9050 | 2.43 | 1.61 | 6.36 | 1.97 | 3.27 | 2.06 | 2 | 2 | detach |
| AimedGun | 6200 | 3.2 | 1.62 | 5.82 | 1.99 | 3.49 | 2.19 | 2 | 2 | attend |
| Pollutio | 9342 | 2.85 | 1.41 | 4.49 | 1.88 | . | . | 2 | 2 | immerse |
| DeadMan | 9433 | 1.84 | 1.19 | 5.89 | 2.6 | 3.37 | 2.16 | 2 | 2 | detach |
| Boys | 9530 | 2.93 | 1.84 | 5.2 | 2.26 | 4.32 | 2.14 | 2 | 3 | attend |
| SickKitt | 9561 | 2.68 | 1.92 | 4.79 | 2.29 | 4.53 | 2.36 | 2 | 3 | immerse |
| ManOnFir | 9635.1 | 1.9 | 1.31 | 6.54 | 2.27 | . | . | 2 | 3 | detach |
| CarAccid | 9920 | 2.5 | 1.52 | 5.76 | 1.96 | 3.09 | 2.13 | 2 | 3 | attend |
| Cow | 9140 | 2.19 | 1.37 | 5.38 | 2.19 | 3.85 | 1.95 | 2 | 3 | immerse |
| Garbage | 9290 | 2.88 | 1.52 | 4.4 | 2.11 | 4.9 | 2.18 | 2 | 3 | detach |
| Skinhead | 9800 | 2.04 | 1.57 | 6.05 | 2.71 | 4.92 | 2.52 | 2 | 3 | attend |
| Injury | 3266 | 1.56 | 0.98 | 6.79 | 2.09 | 2.83 | 2.04 | 2 | 3 | immerse |
| CarAccid | 9911 | 2.3 | 1.37 | 5.76 | 2.1 | 3.54 | 2.13 | 2 | 3 | detach |
| Needles | 9007 | 2.49 | 1.41 | 5.03 | 2.32 | 4.18 | 2.27 | 2 | 3 | attend |
| Seal | 9180 | 2.99 | 1.61 | 5.02 | 2.09 | 4.52 | 2.09 | 2 | 3 | immerse |
| Cemetery | 9000 | 2.55 | 1.55 | 4.06 | 2.25 | 3.25 | 2.13 | 3 | 3 | detach |
| Man | 2190 | 4.83 | 1.28 | 2.41 | 1.8 | 5.92 | 2.01 | 3 | 1 | attend |
| Fingerpr | 2206 | 4.06 | 1.4 | 3.71 | 2.03 | 4.46 | 2.36 | 3 | 1 | attend |
| Chess | 2580 | 5.71 | 1.41 | 2.79 | 1.78 | 5.88 | 1.89 | 3 | 1 | attend |
| FoodBask | 2980 | 5.61 | 1.5 | 3.09 | 1.91 | . | . | 3 | 1 | attend |
| Boat | 5390 | 5.59 | 1.54 | 2.88 | 1.97 | 6.33 | 2.02 | 3 | 2 | attend |
| Mushroom | 5500 | 5.42 | 1.58 | 3 | 2.42 | 6.45 | 2.42 | 3 | 2 | attend |
| RollingP | 7000 | 5 | 0.84 | 2.42 | 1.79 | 6.14 | 2.14 | 3 | 2 | attend |
| Basket | 7010 | 4.94 | 1.07 | 1.76 | 1.48 | 6.7 | 1.48 | 3 | 2 | attend |
| Stool | 7025 | 4.63 | 1.17 | 2.71 | 2.2 | 6.1 | 2.2 | 3 | 3 | attend |
| Baskets | 7041 | 4.99 | 1.12 | 2.6 | 1.78 | . | . | 3 | 3 | attend |
| TrashCan | 7060 | 4.43 | 1.16 | 2.55 | 1.77 | 5.85 | 2.1 | 3 | 3 | attend |
| Bus | 7140 | 5.5 | 1.42 | 2.92 | 2.38 | 5.45 | 1.78 | 3 | 3 | attend |

Note: IAPS pictures were divided into three dimensions: 1- positive emotion; 2- negative emotion; 3- neutral emotion. Pictures listed in the table did not include the neutral pictures that appeared in the first trial of each condition (block).

valmn=valence mean; valsd=valence standard deviation; arsmn=arousal mean; arssd=arousal standard deviation; domlmn=dominance mean; domlsd=dominance standard deviation.
