# Supplementary material for: The Role of Motivation in Cognitive Reappraisal for Depressed Patients
Source: Front Hum Neurosci. 2017 Oct 31;11:516. doi: 10.3389/fnhum.2017.00516 (PMC5671608; doi:10.3389/fnhum.2017.00516)
Supplement: Supplementary file 2 [file Table2.DOC]

**Table S2| Motivation effects on emotion regulation** brain activities during reappraisal

| **Region of activation** | **Side** | **BA** | **MNI Coordinates** | | | **Cluster size** | **Z score** |
| --- | --- | --- | --- | --- | --- | --- | --- |
|  |  |  | **X** | **y** | **z** |  |  |
| 1. **Postive/detach** |  |  |  |  |  |  |  |
| **BASR** |  |  |  |  |  |  |  |
| ***Control>MDD*** |  |  |  |  |  |  |  |
| Lentiform Nucleus | R |  | 16 | -6 | -6 | 26 | 2.18 |
| Midbrain M | L |  | -6 | -4 | -18 | 135 | 2.68 |
|  |  |  |  |  |  |  |  |
| 1. **Negative/detach** |  |  |  |  |  |  |  |
| **A.BASR** |  |  |  |  |  |  |  |
| ***Control>MDD*** |  |  |  |  |  |  |  |
| Midbrain M | L |  | -2 | -14 | -18 | 22 | 2.29 |
| **B.BASF** |  |  |  |  |  |  |  |
| ***Control>MDD*** |  |  |  |  |  |  |  |
| Lentiform Nucleus | R |  | 32 | -14 | -10 | 11 | 1.98 |
|  |  |  |  |  |  |  |  |
| 1. **Positive/immerse** |  |  |  |  |  |  |  |
| **A.BASF** |  |  |  |  |  |  |  |
| ***MDD>Control*** |  |  |  |  |  |  |  |
| Midbrain M | R |  | 16 | -16 | -14 |  | 1.99 |
| **B.BIS** |  |  |  |  |  |  |  |
| ***MDD>Control*** |  |  |  |  |  |  |  |
| Lentiform Nucleus | R |  | 20 | -2 | -6 |  | 1.91 |
|  |  |  |  |  |  |  |  |
| 1. **Negative/immerse** |  |  |  |  |  |  |  |
| **A.BASF** |  |  |  |  |  |  |  |
| ***MDD>Control*** |  |  |  |  |  |  |  |
| Midbrain | R |  | 16 | -16 | -14 |  | 1.99 |
| **B.BIS** |  |  |  |  |  |  |  |
| ***MDD>Control*** |  |  |  |  |  |  |  |
| Lentiform Nucleus | R |  | 20 | -2 | -6 |  | 1.91 |
| Midbrain M | R |  | 14 | -20 | -14 | 86 | 2.66 |

Note: The group-by-motivation interaction identified regions where BIS/BAS scores modulated brain responses differently between the depressed patients and the control group regardless of reappraisal conditions. All clusters were thresholded at *P*<0.05.
